# Expression and migratory analysis of 5 human uveal melanoma cell lines for CXCL12, CXCL8, CXCL1, and HGF

**DOI:** 10.1186/1477-3163-6-2

**Published:** 2007-01-29

**Authors:** Sebastian Di Cesare, Jean-Claude Marshall, Patrick Logan, Emilia Antecka, Dana Faingold, Shawn C Maloney, Miguel N Burnier

**Affiliations:** 1 The Henry C. Witelson Ophthalmic Pathology Laboratory and Registry, McGill University Health Center, Montreal, PQ, Canada; 2 Department of Ophthalmology. Federal University of São Paulo – UNIFESP/EPM. São Paulo, Brazil

## Abstract

**Background:**

The aim of this study was to characterize the presence and roles of CXCL12, CXCL8, CXCL1, and HGF in five human uveal melanoma cell lines, using different methods, in order to ascertain their significance in this disease.

**Methods:**

Five human uveal melanoma cell lines (92.1, SP6.5, MKT-BR, OCM-1, and UW-1) of known proliferative, invasive, and metastatic potential were used in this experiment. A migration assay was used in order to assess the responsiveness of each cell line towards the four chosen chemotactic factors. Immunohistochemistry was then performed for all five cell lines (cytospins) using antibodies directed toward CXCL1, CXCL8 and their receptors CXCR2 and CXCR1 respectively. Quantitative real-time PCR was then performed on all five cell lines in order to establish the presence of these four chemotactic factors.

**Results:**

All five human uveal melanoma cell lines migrated towards the four chosen chemotactic factors at a level greater than that of the negative control. Chemokines CXCL1 and CXCL8 resulted in the greatest number of migrating cells in all five of our cell lines. Immunohistochemistry confirmed the expression of CXCL1, CXCL8, and their receptors CXCR2 and CXCR1 in all five of the cell lines. Quantitative real-time PCR results established expression of CXCL8, CXCL1, and HGF in all 5 cell lines tested. CXCL1 and CXCL8 are highly expressed in SP6.5 and UW-1. None of the five cell lines expressed any detectable levels of CXCL12.

**Conclusion:**

The migratory ability of the 5 human uveal melanoma cell lines was positively influenced by the four chemotactic factors tested, namely CXCL12, CXCL8, CXCL1, and HGF. Self-expression of chemotactic factors CXCL8, CXCL1, and HGF may indicate an autocrine system, which perhaps contributes to the cells' metastatic ability *in vivo*.

## Background

Uveal melanoma is the most common primary intra-ocular tumor in adults. Despite advances in diagnosis and local treatment of patients over the last 30 years, the mortality rate has remained constant, with 30 to 50% of patients developing metastatic disease [1]. Due to the lack of lymphatics in the eye, metastasis of uveal melanoma occurs via hematogenous dissemination, with most metastases developing in the liver [2].

It is now well described and understood that chemo-attractive cytokines referred to as chemokines play a number of functional roles in tumor biology [3]. Chemokines are known to mediate leukocyte trafficking, haematopoiesis, inflammation during infection, chronic inflammatory conditions, angiogenesis, and tumorigenesis [3]. Recent evidence has demonstrated that chemokines play a critical role in cellular transformation, tumor growth, homing, and metastasis [4].

Metastatic disease is typically characterized by initial cell transformation within the primary tumor site followed by angiogenesis, facilitating nutrient delivery to the newly transformed cells. Later events include cellular proliferation and detachment from the local extracellular matrix, followed by cell migration and intravasation into the newly formed angiogenic processes [5]. Interactions between tumor cells and the local environment containing secreted chemokines are thought to be important in a number of tumorigenic processes including the initial cell migration and intravasation; therefore, *in vitro *studies investigating these interactions are important to further gain an understanding of the steps involved in the metastatic cascade.

Members of the CXC chemokine family have been shown to be involved in malignant melanoma cell growth and invasion in an autocrine dependent manner [6]. Chronic over-expression of CXCL1 and CXCL8 in cutaneous melanoma cell lines leads to angiogenesis and cell priming to disseminate from the primary site [7]. Migratory stimulation of uveal melanoma cell lines (SOM196B, SOM157d, SOM267, SOM269) with CXCL1 has been previously shown [8]. Migratory capability of the 5 human uveal melanoma cell lines (92.1, SP6.5, MKT-BR, OCM-1, UW-1) has not yet been established, nor has the auto-expression of these factors within the cells themselves. Due to the extreme differential metastatic abilility of uveal melanoma cell lines 92.1, SP6.5, MKT-BR, OCM-1, UW-1 it is important to investigate how these specific cell lines respond and differentially express these chemo-attractants. Expression of chemokines in primary tumors has previously been shown to promote proliferation and invasion of metastatic cells. The concurrent expression of CXCL12 at distant sites including the lungs, liver, brain and bone marrow suggests a possible homing mechanism for neoplastic cells to these organ specific sites [9].

Due to cytokine involvement in malignant progression of the primary tumor and the metastatic process, chemokines might represent a potential therapeutic target for cancer patients. Promising animal model data has shown that inhibiting interactions between CXCL12 and CXCR4 in a model of murine B16 melanoma cells will impair the metastatic process [10].

Growth factor cytokines were also shown to be implicated into malignant growth of melanoma cells. Hepatocyte growth factor (HGF) has been previously shown to promote the migration of uveal melanoma cell lines *in vitro *(SOM196B, SOM157d, SOM267, SOM269) [8]. Expression of HGF in the 5 human uveal melanoma cell lines (92.1, SP6.5, MKT-BR, OCM-1, UW-1), and the functional significance of this expression has not yet been elucidated.

The present study was designed in order to analyze the expression of CXCL12, CXCL8, CXCL1, and HGF in five human uveal melanoma cell lines (92.1, SP6.5, MKT-BR, OCM-1, UW-1) and to investigate the effects of these factors on the migratory ability of the 5 cell lines. Demonstration of a functional role for CXCL12, CXCL8, CXCL1, and HGF in uveal melanoma may yield novel therapeutic targets. Chemokine receptor inhibitors should be further investigated in the future, in order to possibly delay or inhibit tumor growth and metastasis.

## Methods

### Cell Culture

Five human uveal melanoma cell lines (92.1, SP6.5, MKT-BR, OCM-1, UW-1) were incubated at 37°C in a humidified 5% CO_2 _enriched atmosphere. These cells were cultured with RPMI-1640 medium (Invitrogen), supplemented with 5% heat inactivated fetal bovine serum (FBS; Invitrogen), 1% fungizone (Invitrogen), and 1% penicillin-twice weekly, at every media change, for normal growth by phase contrast microscopy. The cultures were grown to confluence and passaged by treatment with 0.05% trypsin in EDTA (Corning) at 37°C and washed in 7 ml RPMI-1640 media before being centrifuged at 120 g for 10 minutes to form a pellet.

The uveal melanoma cell lines 92.1, SP6.5, MKT-BR, OCM-1, and UW-1 had been established by Dr. Jager (University Hospital Leiden, The Netherlands), Dr. Pelletier (Laval University, Quebec, Canada), Dr. Belkhou (CJF INSERM, France) and Dr. Albert (University of Wisconsin-Madision, USA), respectively [11,12].

### Cell Migration Assay

A QCM™ 24-Well Colorimetric Cell Migration Assay (Chemicon) was used for this experiment. Uveal melanoma cell lines were trypsinized and seeded at a concentration of 1 × 10^6 ^cells/ml in serum free RPMI-1640 medium and placed in the upper well insert of a QCM™ 24-Well Colorimetric Cell Migration Assay (Chemicon). Each individual chemokine was reconstituted and diluted to predetermined, optimum concentrations in serum free RPMI-1640 medium (CXCL12: 100 ng/ml, CXCL8: 300 ng/ml, CXCL1: 40 ng/ml, HGF: 50 ng/ml) and placed in the lower chamber of the cell migration assay. All five cell lines were done in triplicate for each of the selected chemo-attractants. The 24-Well Plates were then incubated for 24 hours in a humidified 5% enriched CO_2 _atmosphere. Cells that migrated through the 8 μm pore membranes, located at the bottom of every well insert, were stained and eluted. The optical density of the stained cells was then read by a colorimetric plate reader at 560 nm.

### Immunohistochemistry

Cytopsins of the 5 human Uveal Melanoma cell lines were made using a Cytospin3 machine (Shandon). Cells from culture were diluted to a concentration of 250,000 cells/ml, and a 300 μL solution at that concentration was placed in each spin to be evenly plated on each slide. All slides were then immunostained with primary anti-human monoclonal antibodies against CXCL1 (clone #:20326), CXCL8 (clone #: 6217), and their receptor CXCR1 (clone #: 42705), and CXCR2 (clone #: 48311) using a standard Avidin-Biotin Complex method for each antibody (R&D Systems).

### Quantitative real-time PCR

Appropriate primers for CXCL12, CXCL8, CXCL1, and HGF were chosen, (QuantiTect^® ^Primer Assays) for use with QuantiTect SYBR^® ^green kits (Qiagen), for this experiment. Total mRNA was then extracted from all 5 human uveal melanoma cell lines from culture (92.1, SP6.5, MKT-BR, OCM-1, UW-1) using an RNeasy RNA extraction kit (Qiagen). A Chromo4 thermocycler (MJ Research) was used for all experiments and all results were analyzed using the GeneEx software. Beta actin was used as a housekeeper gene for purposes of normalization.

### Statistical Analysis

Results from the migration assay for each cell line were directly compared to the negative control using a Student's T-test.

## Results

### Migration Assay

**Figure 1:**
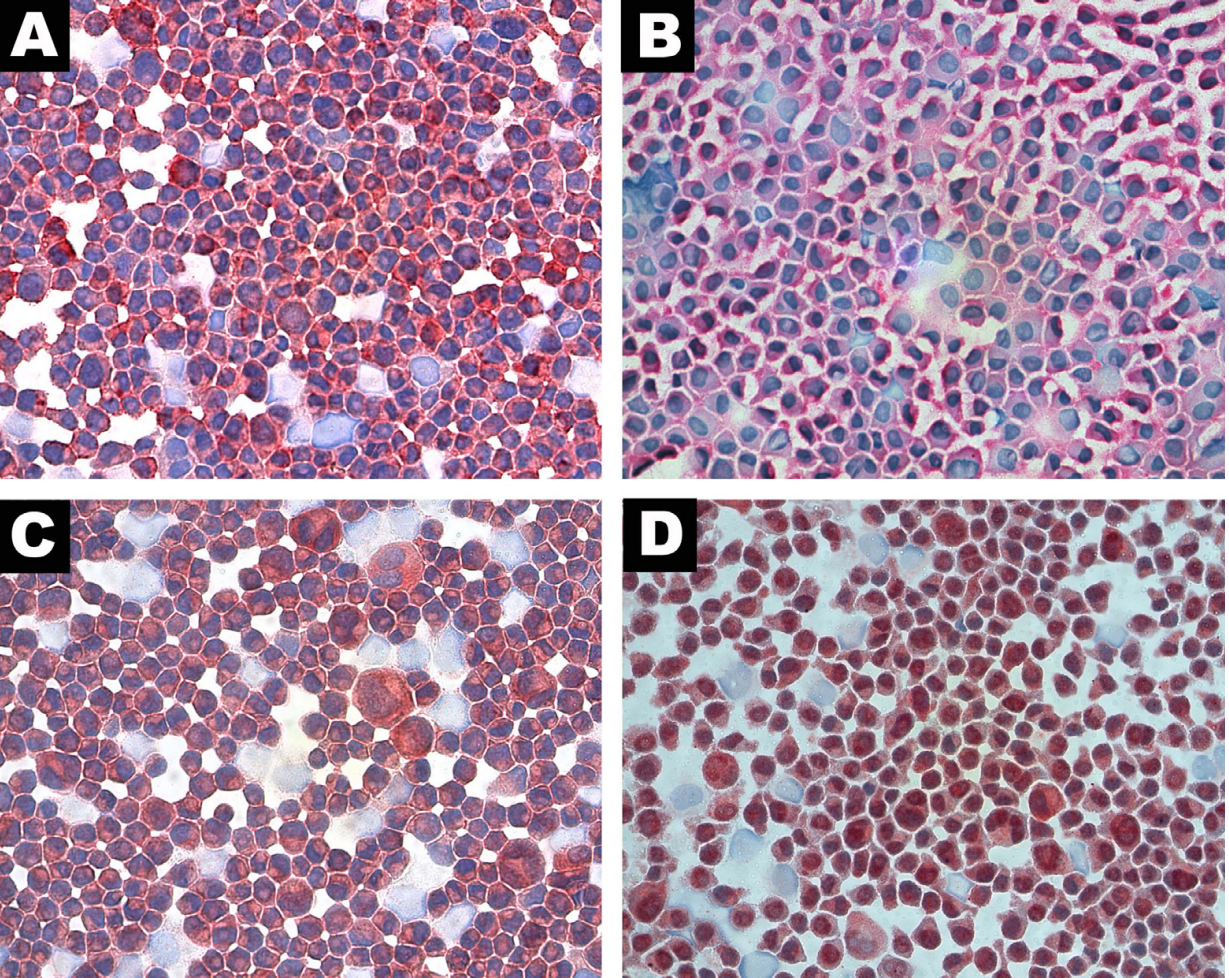
**Photomicrographies. Cytospins of uveal melanoma cell lines**. (A) UW-1 staining positive for CXCL1 (B) UW-1 staining positive for CXCR2 (C) 92.1 staining positive for CXCL8 (D) OCM-1 staining positive for CXCR1 (red chromogen, ×400).

All 5 human uveal melanoma cell lines (92.1, SP6.5, MKT-BR, OCM-1, UW-1) migrated towards the selected chemo-attractants at a level greater than the selected negative control (serum-free RPMI media) (p < 0.05) except for cell line OCM-1 towards chemokine CXCL1 (p-value = 0.0575). Chemokines CXCL1 and CXCL8 resulted in the greatest average migration for all five cell lines followed by CXCL12 and HGF respectively. (Figure 1)

### Immunohistochemistry

**Figure 2:**
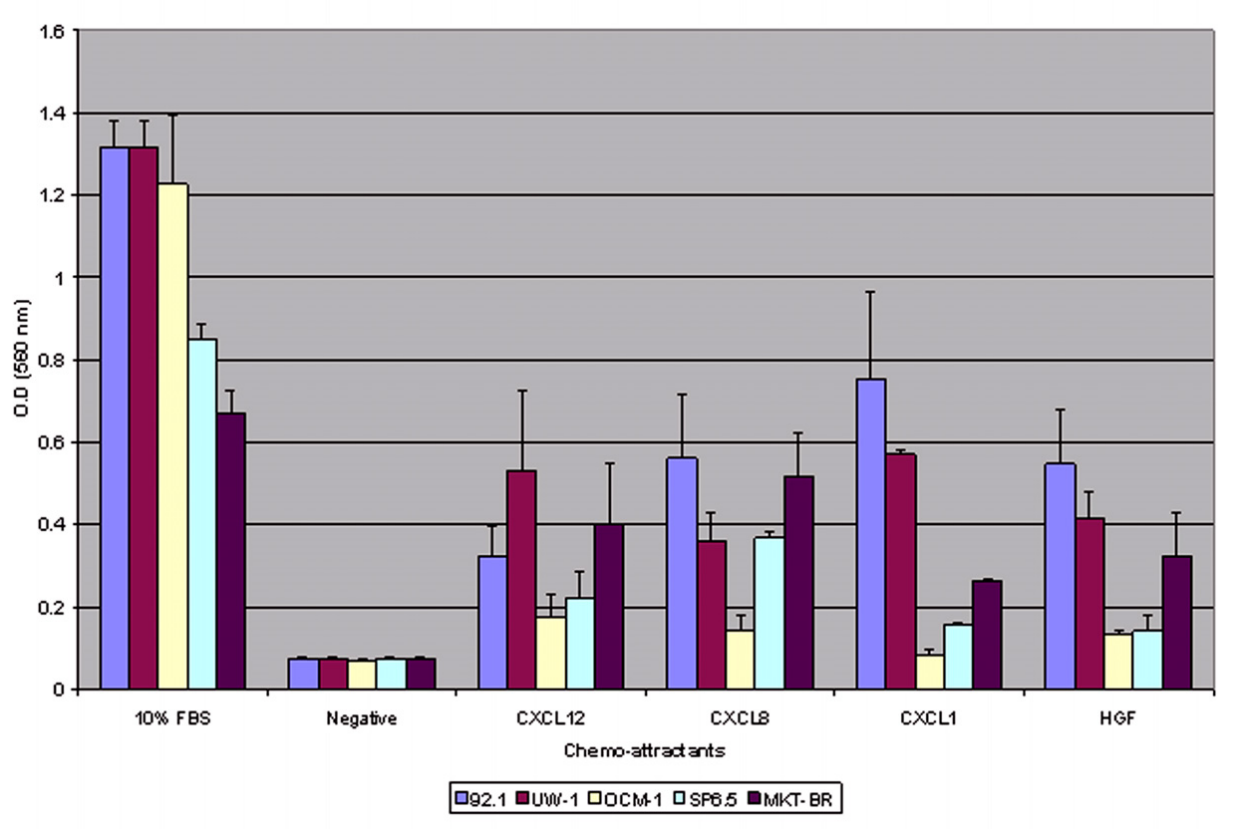
Cell migration data for 5 human uveal melanoma cell lines.

All 5 human uveal melanoma cell line cytospins stained positive for CXCL1, CXCL8, and their receptors CXCR2 and CXCR1 respectively. The ligand staining for CXCL1 and CXCL8 showed more of a cytoplasmic stain while the receptor staining for CXCR1 and CXCR2 showed more membranous staining when viewed at a magnification of 400×. (Figure 2)

### Quantitative Real-Time PCR

**Figure 3:**
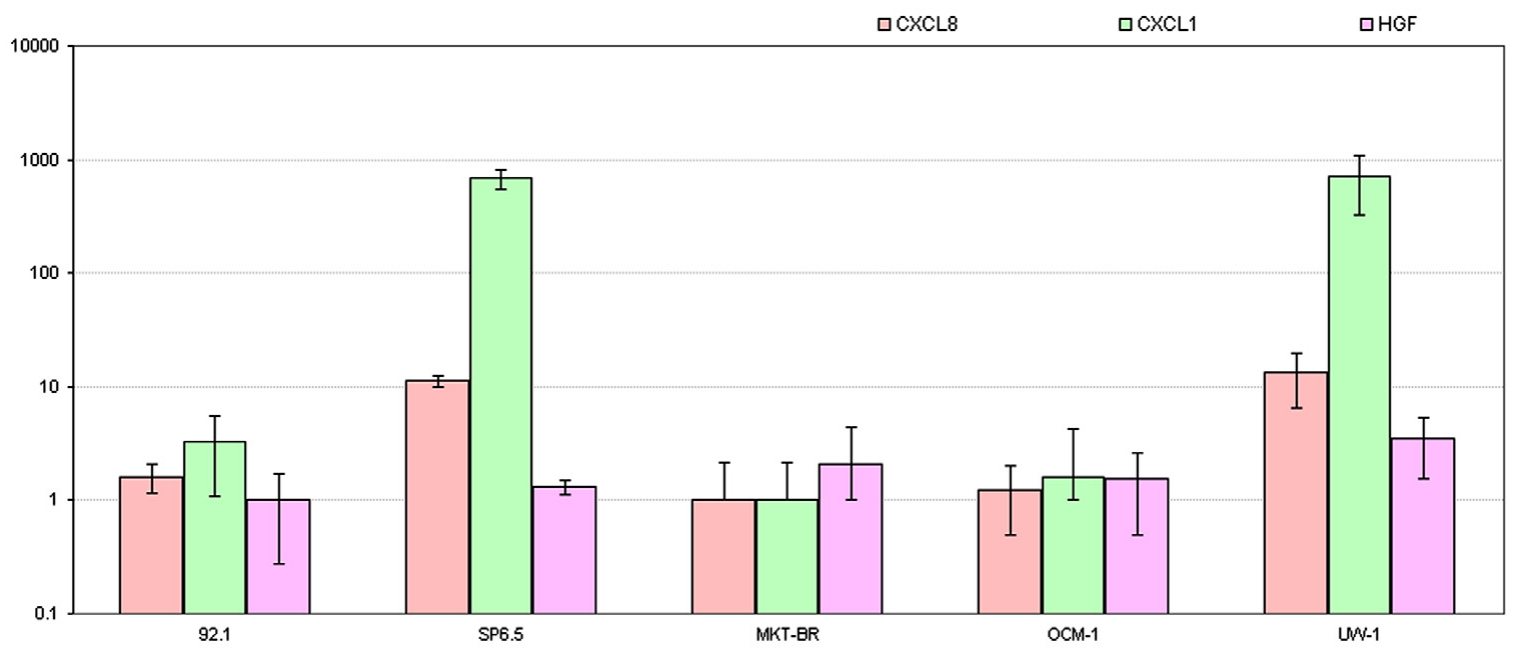
**Quantitative real-time PCR graph**. Total mRNA extracted from 5 human uveal melanoma cell lines amplified for CXCL8, CXCL1, and HGF primers displaying different relative expression.

All 5 human uveal melanoma cell lines were shown to produce significant levels of CXCL8, CXCL1, and HGF. SP6.5 and UW-1 cell lines exhibited the greatest production of CXCL1 and CXCL8 followed by 92.1 > OCM-1 > MKT-BR. None of the 5 cell lines expressed detectable levels of CXCL12. (Figure 3)

## Discussion

The prominent site of metastatic disease in uveal melanoma patients is the liver. Many reasons for this organ-specificity still remain unknown. Recent studies in other related cancers have shown that chemokines and cytokines play an integral role in "guiding" these cells to organ specific locations. For example, malignant cutaneous melanoma and breast carcinoma express chemokine receptors which may play an important role in determining the metastatic location of these tumor cells [8]. More specifically, recent evidence suggests that the CXCR4/CXCL12 axis plays an integral role in guiding metastatic cells from breast carcinoma [13].

The five human uveal melanoma cell lines used in this study have been previously characterized and classified by their different metastatic and proliferative potential [14]. From this previous study, it was shown that the cell lines 92.1 and SP6.5 were classified as being highly proliferative and having high metastatic potential relative to the other three cell lines tested (MKT-BR, OCM-1, UW-1).

In our study, the cell line SP6.5 also expressed the highest levels of CXCL1 and CXCL8 as measured by real-time PCR analysis. These chemokines have previously been shown to stimulate melanoma cellular proliferation [15] and CXCL1 and CXCL8 were shown to be involved in invasion and metastasis of melanoma cells [9]. CXCL8 has been linked to migration of melanoma cells [16] and the induction of matrix metalloproteinase-2 expression, facilitating extracellular matrix degradation and tumor cell migration [17].

In contrast with the results obtained for SP6.5, the MKT-BR cell line showed very low levels of expression of CXCL8, CXCL1, and HGF. This cell line has been previously characterized as having no metastatic potential in an animal model [18]. It may be possible that these specific factors are needed in higher concentration in order to achieve a higher metastatic potential.

It was previously suggested that CXCL12 expression at distant sites including the lungs, liver, brain and bone marrow may contribute to a possible homing mechanism for neoplastic cells to these organ-specific sites. In our study, none of the 5 human uveal melanoma cell lines expressed any detectable levels of CXCL12. The only known receptor for CXCL12, CXCR4, was previously shown to be expressed in different cancers including cutaneous melanoma, colon cancer, prostate cancer, breast cancer and neuroblastoma [19]. Unpublished data from our laboratory has also shown that CXCR4 was positive in 31 primary human uveal melanoma patient samples. It is possible that CXCR4 positive cells will migrate towards organs secreting CXCL12 (bone, liver, lung, brain) which display organ specific homing and metastasis [19]. In uveal melanoma, the pattern of metastatic spread is predominantly to the liver, therefore suggesting that CXCL12 expression in the liver may be involved in the homing of uveal melanoma cells to this site.

Hepatocyte growth factor (HGF) initiates invasive oncogenic cellgrowth [20]. HGF initiates internal cellular signaling to the cancer cells, which allows the cells to invade and metastasize by combining cell proliferation, motility, morphogenesis, and cell survival [20]. In uveal melanoma, the epitheliod cells, (the cell type with the highest metastatic potential) were shown to express the c-met receptor for HGF [21]. It is possible that the expression of HGF by these 5 cell lines does induce the motility of these c-met positive cells, which can also be homed to the liver via HGF secretion.

## Conclusion

In conclusion, characterizing the expression of CXCL12, CXCL8, CXCL1, and HGF in uveal melanoma cell lines leads us to a better understanding of how these cytokines are integral in the metastatic process. Further characterization and understanding of their roles may lead to novel therapies that target these factors, which may delay or inhibit the metastatic process.

## Competing interests

The author(s) declare that they have no competing interests.
